# Clinical Outcomes of Bovine Bone Xenografts Following Sequestrectomy in Advanced Medication-Related Osteonecrosis of the Jaw

**DOI:** 10.3390/dj14020123

**Published:** 2026-02-21

**Authors:** Raluca Maracineanu, Ciprian Roi, Marilena Dinuti, Alexandra Roi, Florin Urtila, Anca Tudor, Ivona Mihaela Hum, Serban Talpos-Niculescu

**Affiliations:** 1 Doctoral School, “Victor Babeș” University of Medicine and Pharmacy, 300062 Timisoara, Romania; raluca.zibileanu@umft.ro; 2 Department of Anesthesiology and Oral Surgery, Research Center of Dento-Alveolar Surgery, Anesthesia and Sedation in Dental Medicine, “Victor Babes” University of Medicine and Pharmacy, Eftimie Murgu Sq. No. 2, 300041 Timisoara, Romania; ciprian.roi@umft.ro; 3 Department of Biochemistry and Pharmacology, Discipline of Biochemistry, Victor Babeș” University of Medicine and Pharmacy, 300041 Timisoara, Romania; motoc.marilena@umft.ro; 4 Department of Oral Pathology, Multidisciplinary Center for Research, Evaluation, Diagnosis and Therapies in Oral Medicine, “Victor Babes” University of Medicine and Pharmacy, Eftimie Murgu Sq. No. 2, 300041 Timisoara, Romania; alexandra.moga@umft.ro; 5 Discipline of Oral and Maxillofacial Surgery, Faculty of Dental Medicine, “Victor Babeș” University of Medicine and Pharmacy, 300062 Timisoara, Romania; urtila.florin@umft.ro (F.U.); talpos.serban@umft.ro (S.T.-N.); 6 Department of Functional Sciences, “Victor Babeș” University of Medicine and Pharmacy, 300041 Timisoara, Romania; atudor@umft.ro

**Keywords:** MRONJ, bisphosphonates, zolendronic acid, bone augmentation, xenograft, Bio-Oss

## Abstract

**Background/Objectives**: Bisphosphonates, a class of drugs that are widely used in the treatment of neoplastic diseases, can lead to the development of medication-related osteonecrosis of the jaw (MRONJ). This condition is challenging to manage due to the high incidence of postoperative complications: superinfections, local wound dehiscence, or fractures in pathological bone. The aim of this study is to evaluate the therapeutic role of bovine-derived xenografts in the management of MRONJ. **Methods**: This retrospective observational study evaluates the clinical outcomes of patients with confirmed stage II or III MRONJ, after surgical treatment with Bio-Oss application. All patients had received zoledronic acid therapy, which was discontinued for a minimum of four months prior to surgical intervention. The surgical protocol included local debridement, sequestrectomy, and grafting of the residual defect with a bone substitute, followed by periodic clinical evaluations and monitoring of local healing with a follow-up period of up to one year. **Results**: Of the total number of patients treated according to this surgical protocol, 85.71% achieved favorable healing without complications at 8 weeks. Cases with poor local healing results were more likely to have prolonged zoledronic acid administration. **Conclusions**: Within the limits of this retrospective observational study, the use of bovine-derived xenografts following sequestrectomy in stage II–III MRONJ was associated with satisfactory local healing in several cases. However, considering the limited sample size and lack of a comparator group, these findings should be interpreted cautiously. To better understand the connection between the length of antiresorptive therapy, surgical management techniques, and postoperative outcomes, more prospective, multicenter trials with bigger patient cohorts are needed.

## 1. Introduction

Bisphosphonates are a class of drugs that are used in various diseases to prevent bone loss. Their main role is to inhibit the activity of osteoclasts, so they can be easily bound to each bone surface being accumulated in the bone matrix. Osteoclasts ingest these bisphosphonates during the bone remodeling process, acting as an isoprenoid diphosphate lipid and preventing osteoclast death [1]. They are used in the treatment of various diseases such as tumor pathology with bone metastases, Paget’s disease, multiple myeloma, and in the treatment of osteoporosis or osteogenesis imperfecta [2]. The first studies on the use of biphosphonates in humans were conducted in 1969 by Herbert Fleisch, when Didronel (etindronate) was used to treat a case of myositis ossificans [3]. Several types of bisphosphonates subsequently appeared on the market, administered orally, or intravenously much more potent than Didronel. For the treatment of osteoporosis, the most used are Fosamax (alendronate), Actonel (risedronate), and Bonviva (ibandronate) with oral administration, and the most powerful of them, Aclasta (zoledronic acid), is administered intravenously at 5 mg once a year. For the treatment of bone metastasis, Aredia (disodium pamidronate) and Zometa (zolendronate) are the drugs of choice and are used intravenously every 3 weeks [4,5]. Zolendronic acid is thought to be 10,000-times more potent than Didronel. The bone half-life of bisphosphonates is considered to be more than 11 years [6].

All drugs from the bisphosphonate class have a side effect on the jawbones by accumulating in excessive amounts due to the increased turnover of these bones [7]. Medication-related osteonecrosis of the jaw (MRONJ) can develop as a result of necrosis caused by a variety of traumatic mechanical causes affecting the maxillary bone when bisphosphonates are present [8]. The same deleterious effect on maxillary bones, causing MRONJ, can also be caused by Denosumab, a monoclonal antibody used in the treatment of bone metastases [9].

The treatment strategies for MRONJ include conservative therapies or surgical approaches, and are applied depending on the staging of the osteonecrosis. However, postoperative complications, like dehiscences and local superinfections, are quite common [10].

Various surgical adjuvant methods have been attempted to promote tissue and bone healing, such as tension-free coverage of defects with local mucosal flaps [11], local application of growth factors like PRF [12,13,14,15], ozone application [16], laser therapy [17], or local application of stem cells [18], with results that have been more or less favorable.

The osteogenic and osteoconductive benefits of bone substitute materials in the regeneration of hard tissue defects in the maxillary bones are well-documented in the scientific literature. Among naturally derived grafts, xenografts are the most widely utilized, primarily due to their excellent biocompatibility and osteoinductive properties [19].

However, their application in medication-related osteonecrosis of the jaw (MRONJ) remains insufficiently investigated, with current evidence limited to a small number of animal studies and isolated clinical case reports [20]. Therefore, despite the modest number of cases, we aimed to offer this retrospective exploratory inquiry due to the poor scientific information on the application of xenografts and the scarcity of published data supporting their potential benefits in MRONJ treatment.

There are some recent studies that have demonstrated that synthetic materials, such as calcium phosphate (CP) and beta-tricalcium phosphate (β-TCP), may possess the ability to absorb and neutralize zoledronic acid within the osseous matrix [21].

In light of these findings, we consider it clinically relevant to present this retrospective study involving the use of bovine-derived xenografts (Bio-Oss) following the surgical removal of necrotic bone in confirmed MRONJ cases, with the aim of evaluating their potential therapeutic and preventive roles in the management of this pathology.

## 2. Materials and Methods

This is a retrospective observational study evaluating the clinical outcomes of 14 patients diagnosed with MRONJ, who underwent a specific surgical treatment, rarely reported in the scientific literature, involving the application of a xenograft, specifically bovine bone substitute, between January 2018 and January 2025 at the Oral and Maxillofacial Surgery Clinic in Timișoara. Data were collected from electronic medical records and patient charts, including demographic variables, associated medical conditions, surgical details and procedures, postoperative complications, and healing time.

Ethical approval for the publication of this research was obtained, prior to data collection and analysis, from the Independent Ethics Committee of the “Victor Babeș” University of Medicine and Pharmacy, Timișoara, Romania (approval no. 51/2 October 2023) and also from the Timișoara Municipal Clinical Emergency Hospital (approval no. E-892/25 February 2025).

Inclusion criteria encompassed patients receiving zoledronic acid for various neoplastic diseases, with the exception of those treated for osteoporosis or multiple myeloma. There were no age or comorbidity-related limitations on the inclusion of male or female patients. The present study included exclusively stage II and stage III MRONJ cases, in which surgical management represents the treatment of choice.

The exclusion criteria included the absence of osteonecrotic lesions in the mandibular or maxillary regions, the absence of previous zoledronic acid administration, a history of radiotherapy affecting the facial bones, the presence of malignant pathology in the head and neck region, and continuous zoledronic acid therapy that could not be stopped for at least four months. Additional exclusion criteria comprised pregnant or breastfeeding women and pediatric patients.

The surgical procedures included sequestrectomy with the removal of necrotic bone, local antiseptic irrigation, debridement of necrotic soft and hard tissues, and local grafting defect using a xenograft—specifically bovine-derived Bio-Oss (Geistlich)—followed by tension-free flap closure.

All patients included in this study had been undergoing long-term treatment with zoledronic acid prescribed for malignant tumor pathology. This treatment was discontinued for a minimum of 4 months prior to the surgical intervention. Initial diagnostic evaluation consisted of cone beam computed tomography (CBCT) or panoramic radiography to confirm stage II or III MRONJ and to identify the presence of bone sequestration or areas of osteonecrosis.

All surgical procedures were performed under local anesthesia using 4% articaine with epinephrine at 1:100,000 (Ubistesin Forte, 3M ESPE, 3M Healthcare, Seefeld, Germany). The surgical field was disinfected both extraorally (skin disinfection) with a 100 mg/mL povidone–iodine solution and intraorally with a 0.2% chlorhexidine solution, followed by isolation of the operative area.

**Figure 1:**
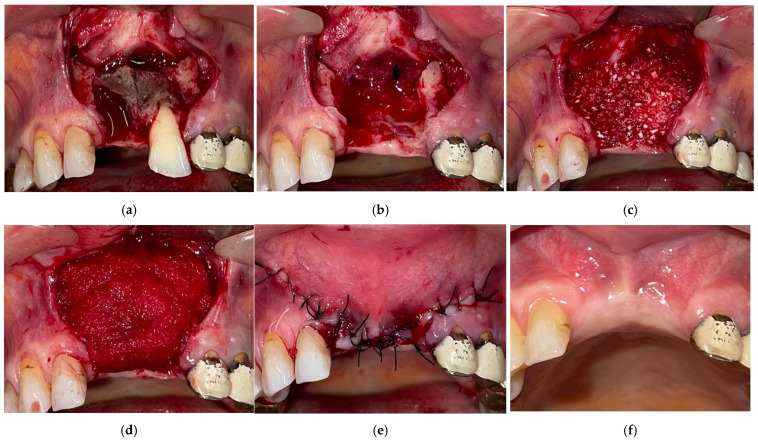
The surgical protocol followed in stage II or III MRONJ: (**a**) anterior maxillary osteonecrosis with the presence of a bony sequestrum; (**b**) residual bony cavity following the removal of the necrotic bone; (**c**) grafting of the defect with bovine bone (Bio-Oss^®^, Geistlich, Wolhusen, Switzerland); (**d**) placement of a protective membrane (Mucograft^®^, Geistlich) covering the grafted site; (**e**) defect closure using a locally advanced tension-free flap followed by suturing; (**f**) local healing at 8 weeks.

To ensure complete exposure of the necrotic bone region and to enable tension-free primary closure, wide vestibular mucogingival flaps were created using sulcular and releasing incisions. The flaps were subsequently mobilized through periosteal scoring to reduce tension. The cleavage plane between necrotic and vital bone was identified, and the bone sequestrum was completely removed using a dental luxator (Figure 1a,b). Teeth presenting marked mobility or integrated within the sequestrum were also extracted. The margins of the resulting bone defect were regularized using a bone rongeur.

In cases where a well-demarcated sequestrum was not clinically evident, necrotic bone tissue was removed using a bone rongeur or round burs mounted on a straight handpiece under continuous irrigation with sterile 0.9% NaCl solution.

The resultant bone defect was filled with bovine-derived cancellous bone substitute granules (Bio-Oss, Geistlich; 1–2 mm) (Figure 1c). The grafted area was then covered with a resorbable collagen membrane (Bio-Gide, Geistlich) and secured in place with titanium pins or resorbable sutures to promote guided bone regeneration and maintain the stability of the bone granules (Figure 1d). The flap was then repositioned without tension and sutured using non-resorbable Supraamid 4/0 sutures (B. Braun). Sutures were removed 10 days postoperatively (Figure 1e,f).

All patients underwent antibiotic therapy after the surgery with broad-spectrum amoxicillin–clavulanic acid (1000 mg/200 mg) administered every 12 h for seven days, accompanied by analgesics and non-steroidal anti-inflammatory drugs. Removed bone fragments were submitted for histopathological examination to confirm the diagnosis of MRONJ.

Follow-up was performed at 4 and 8 weeks postoperatively, and subsequently every 3 months up to one year. In the present study, we considered the outcomes obtained at the 8-week follow-up as well as at the last available follow-up visit, which was either at 9 or 12 months depending on the individual case, as not all patients attended the 1-year follow-up and, in some cases, the final assessment was conducted at 9 months. The absence of documented local wound dehiscence or superinfection in the associated medical records at the matching follow-up appointment was considered favorable healing. Radiological follow-up using CBCT or panoramic radiographs was also performed to assess postoperative bone healing and regeneration. However, imaging tests were not used as a criterion for evaluating local healing in the current study since they were not consistent across all patients. In any case, MRONJ is defined in the literature by the absence of local dehiscence or superinfection at the 8-week follow-up; therefore, imaging evaluation is not considered a diagnostic criterion for this condition.

## 3. Results

Despite being done on a relatively small cohort of 14 cases, we consider the current statistical analysis to be meaningful given the small number of cases treated in accordance with the surgical protocol previously described. This is supported by the fact that, to our knowledge, no studies in the existing literature report larger statistical series for such treatment. Most available research consists of case reports or, at most, animal studies; therefore, we regard the statistical evaluation carried out in the present study as both relevant and timely, even though it is based on a limited number of cases.

The statistical analysis was performed using JASP v0.19.3 (open source statistical analysis software supported by the University of Amsterdam). Quantitative variables were expressed as mean ± standard deviation and as median (Quartile1–Quartile3). The type of distribution of continuous variables was assessed using the Shapiro–Wilk test to determine the normality of the numerical data distribution. The comparison between two independent groups was made using the Mann–Whitney U Test. The nominal variables were described as number and percentage and the association between the two was made using the Chi2 Test. The results are considered significant for a value of *p* < 0.05.

Among the 14 patients included in the present study, there were 8 females and 6 males, corresponding to a proportion of 57.1% females and 42.9% males. This gender distribution showed no statistically significant correlation with the duration of zoledronic acid administration or the occurrence of postoperative complications. The mean age of the patients was 65.9 years, ranging from 43 to 81 years.

Of the 14 cases of medication-related osteonecrosis of the jaw (MRONJ), 9 were located in the mandible and 5 in the maxilla. No statistically significant correlation was observed between the site of osteonecrotic lesions and the cases presenting poor local healing; the two cases with local complications were located in the maxilla and the mandible, respectively. The proportion of MRONJ stage II cases was 64.3%, while stage III cases accounted for 35.7%. Although stage II was numerically more frequent, the difference was not statistically significant relative to the sample size (*n* = 14) (*p* > 0.05).

Of the total number of cases treated according to the aforementioned surgical protocol, 85.71% achieved favorable outcomes, with no postoperative complications. The absence of complications was defined as complete tissue healing without wound dehiscence or local secondary infection at 8 weeks postoperatively (Table 1).

We also performed a follow-up evaluation at approximately one year (a period between 9 and 12 months was considered) and only one of the 14 patients continued to present a local dehiscence at the site of bovine bone grafting, accompanied by clinical signs of secondary infection and the development of new MRONJ lesions. This patient was also the one with the longest duration of zoledronic acid therapy (44 months).

For all patients included in the present study, the discontinuation period of zoledronic acid therapy, prior to surgical intervention, was more than four months. The mean drug holiday in patients who developed postoperative complications was seven months, whereas in those without complications, it was approximately five and a half months. No significant association was found between the length of the drug holiday and the occurrence of postoperative complications (Mann–Whitney U test, *p* = 0.185). We performed logistic regression with the dependent variable complication (yes/no) and the independent variables drug holiday (month) and zolendronic acid duration (month), resulting in insignificant associations.

**Figure 2:**
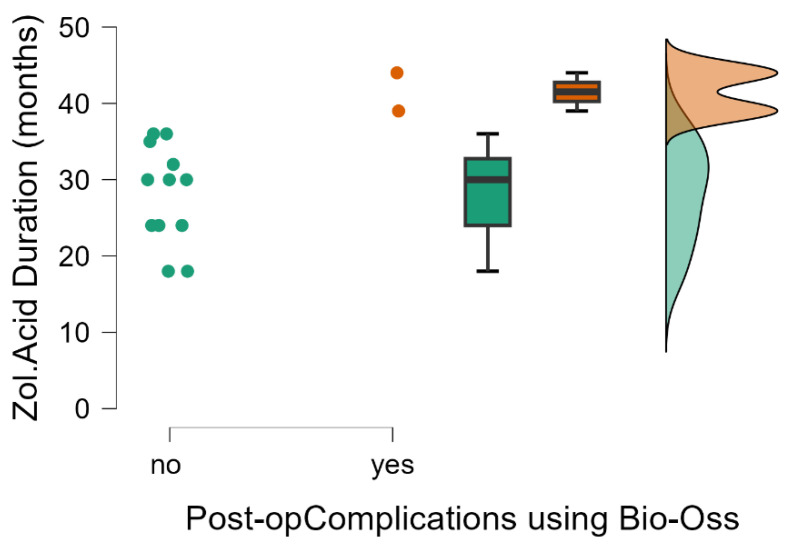
Postoperative complications associated with the use of Bio-Oss in relation to the duration of antiresorptive therapy with zoledronic acid.

The mean duration of bisphosphonate therapy prior to its discontinuation for surgical intervention was two and a half years. We performed a statistical comparison between the duration of zoledronic acid intake in patients with favorable outcomes at 8 weeks after surgery and those with unfavorable outcomes. It was concluded that in patients who developed postoperative complications, the duration of bisphosphonate therapy administered cumulatively was significantly longer than in those with favorable outcomes (Mann–Whitney test, *p* = 0.034) (Table 2 and Table 3) (Figure 2). We performed a power analysis (G*Power, Mann–Whitney test, two-tailed), which indicated that, for n1 = 12 and n2 = 2, with α = 0.05 and 80% power, this study is vulnerable only to very large effects (effect size ≈ 1.8). This suggests that the observed differences are compatible with a large effect size, but the very small size of the second group strongly limits the generalizability of the results, which should be considered exploratory.

At the one-year follow-up, considering that only one case with an unfavorable outcome was identified, no statistical comparison could be made between the duration of zoledronic acid administration or the length of the preoperative drug holiday and the clinical evolution.

## 4. Discussion

The use of bone substitute materials for grafting defects that results from sequestrectomy in patients diagnosed with MRONJ remains relatively underexplored in the current scientific literature. Several studies made on animal models have reported the beneficial effects of synthetic bone substitutes, such as beta-tricalcium phosphate (β-TCP), calcium phosphate, and grafts incorporating growth factors, including bone morphogenetic protein-2 (BMP-2) and transforming growth factor-beta (TGF-β). In a study from 2021, conducted on eighteen male mice, MRONJ was experimentally induced through the administration of zoledronic acid over a period of five weeks. The control group, which did not receive any bone grafting material, demonstrated a bone regeneration rate of 58%, compared to 66% in the experimental group treated with beta-tricalcium phosphate (β-TCP) and 59% in the group receiving bovine-derived xenograft. Also, the histological analysis revealed a lower incidence of bone lacunae in the groups treated with bone regenerative materials [22]. Although in our study the outcome after the application of the bovine xenograft was only 1% better, the results should not necessarily be interpreted as optimistic, but rather as indicating a new research direction for the treatment of this particularly challenging and refractory condition, MRONJ.

A recent animal study conducted on rats evaluated the preventive effect of Bio-Oss application in the mandibular molar post-extractional sockets, both alone and in combination with platelet-rich plasma (PRP). The rate of bone exposure was 83.33% in the control group, 66.67% in the group treated with Bio-Oss alone, and 0% in the group treated with the Bio-Oss/PRP combination. The study found that using bovine-derived xenografts like Bio-Oss, either by itself or in combination with growth factors like PRP, encourages local post-extraction healing and successfully stops animals from developing medication-related osteonecrosis of the jaw (MRONJ) [20]. These animal studies provide an important background for understanding the potential effects of xenografts on bone healing. In the present study, we observed that the use of bovine bone was associated with favorable local healing outcomes and a lower incidence of recurrence and postoperative complications. To date, no similar studies have been conducted in humans, and it is important to note that these findings should be interpreted as preliminary observations within our retrospective cohort.

The actual literature is still divided regarding the surgical treatment of MRONJ at all stages of the disease, but several studies show that surgical therapy has greater success rates than conservative treatment. In 2017, Eguchi T. reported an 89.3% success rate for surgical treatment, which was significantly superior to non-surgical management (33.3%) [23]. Similarly, Osaka R. et al., in a retrospective study from 2021, concluded that surgical removal of necrotic bone is clearly recommended for stage II and III MRONJ with better postoperative results [24]. El-Rabbany et al. conducted a retrospective cohort study in 2019, reporting disease resolution in 70% of MRONJ cases treated with surgical therapy [25]. In the present study cohort, a rate of 85.71% favorable outcomes was observed following the use of bovine bone substitution materials, representing the outcomes achieved within this group and providing contextual insight into the effects of this treatment approach.

Prolonged use of monoclonal antibody or bisphosphonate therapy makes surgical treatment more challenging in MRONJ cases and raises the likelihood of postoperative complications and local recurrence. Multiple specialized studies reveal that one of the most important risk factors for MRONJ recurrence after surgical therapy is the duration of prior antiresorptive medication use, with treatment extending beyond 18 months being a negative prognostic factor [26,27,28,29]. In the present study, a longer duration of zoledronic acid administration appeared to be more common among cases with postoperative complications than among those with favorable outcomes.

There are also several case reports in the literature describing the potential role of guided bone regeneration after sequestrectomy in patients receiving antiresorptive medication, through local grafting with xenografts aimed to accelerate local healing. The local results obtained in these cases were satisfactory, with no occurrence of local dehiscence or secondary infections. However, the lack of larger patient samples and large-scale studies in the studies published in the scientific literature, indicates that the therapeutic approach involving the use of xenografts in patients diagnosed with MRONJ is not yet a routine practice in everyday clinical settings [30,31].

## 5. Conclusions

In our cases, the use of bone xenografts, specifically bovine bone, for grafting local mandibular or maxillary defects following sequestrectomy in MRONJ lesions was observed to be associated with favorable local healing outcomes. These observations suggest a potential positive role of Bio-Oss in reducing postoperative complications. However, these findings should be interpreted with caution, as they are based on a retrospective analysis with a limited sample size, and do not allow for definitive clinical recommendations.

Additionally, our results indicate a possible association between the duration of antiresorptive medication administration and local healing outcomes. Prolonged exposure to zoledronic acid was more frequently observed among patients who developed postoperative complications following surgical treatment of stage II or III MRONJ. This observation should be considered exploratory and warrants further investigation in larger, prospective studies.

The present study has several limitations that should be acknowledged. First, the observational retrospective design of this study inherently limits the control over data collection and standardization of the evaluated parameters. In addition, the sample size was very small, including only 14 cases, and the group of patients who experienced postoperative complications consisted of only 2 cases, which severely limited the reliability of the statistical comparisons performed. Furthermore, not all patients benefited from a standardized radiological follow-up, and different imaging modalities were used to assess healing, potentially introducing variability in outcome evaluation. The final follow-up was performed at variable time points ranging from 9 to 12 months, without a clearly defined and uniform follow-up period. While the results of our study appear promising, and local healing following the use of bovine bone was generally favorable, we acknowledge that spontaneous healing or the surgical procedure itself may also have contributed to the observed outcomes. Moreover, there are very limited data available in the current literature addressing similar therapeutic approaches, with existing evidence mainly consisting of isolated case reports, which prevents a robust comparison of our results with previously published studies. Although inclusion and exclusion criteria were predefined, the retrospective design and small subgroup sizes may limit generalizability and introduce potential selection bias.

Through the present study, we aimed to provide descriptive clinical observations on the management of MRONJ cases and emphasizes the need for future multicenter, prospective studies with larger cohorts, standardized imaging protocols, and clearly defined follow-up intervals to further explore the potential benefits and reproducibility of the proposed approach.

## Figures and Tables

**Table 1:** Frequency of post-op complications using Bio-Oss.

Post-Op Complications Using Bio-Oss	Frequency	Percent	Valid Percent	Cumulative Percent
no	12	85.71	85.71	85.71
yes	2	14.29	14.29	100
Total	14	100		

**Table 2:** The occurrence of postoperative complications in relation to the total duration of zoledronic acid therapy.

Group Descriptives								
	Post-op complications	N	Mean	SD	SE	Coefficient of variation	Mean Rank	Sum Rank
Zol.Acid Duration (months)	no	12	28.083	6.417	1.852	0.228	6.5	78
	yes	2	41.5	3.536	2.5	0.085	13.5	27

**Table 3:** Duration of bisphosphonate therapy administered cumulatively was significantly longer than in those with favorable outcomes (Mann–Whitney test, *p* = 0.034).

Independent Samples T-Test			
	U	df	*p*
Zol.Acid Duration (months)	0		0.034

*Note.* Mann–Whitney U test.

## Data Availability

The data presented in this study are available on request from the corresponding authors. The data are not publicly available due to restrictions related to the privacy of the patients and the funding protocol.

## Abbreviations

The following abbreviations are used in this manuscript:

MRONJ	Medication-related osteonecrosis of the jaw
PRF	Platelet-rich fibrin
CP	Calcium phosphate
CBCT	Cone beam computed tomography
β-TCP	Beta-tricalcium phosphate

## References

[1] Munoz M.A., Fletcher E.K., Skinner O.P., Jurczyluk J., Kristianto E., Hodson M.P., Sun S., Ebetino F.H., Croucher D.R., Hansbro P.M. (2021). Bisphosphonate drugs have actions in the lung and inhibit the mevalonate pathway in alveolar macrophages. eLife.

[2] Drake M.T., Clarke B.L., Khosla S. (2008). Bisphosphonates: Mechanism of action and role in clinical practice. Mayo Clin. Proc..

[3] Fleisch H., Russell R.G., Francis M.D. (1969). Diphosphonates inhibit hydroxyapatite dissolution in vitro and bone resorption in tissue culture and in vivo. Science.

[4] Russell R.G., Croucher P.I., Rogers M.J. (1999). Bisphosphonates: Pharmacology, mechanisms of action, and clinical uses. Osteoporos. Int..

[5] Chern B., Joseph D., Joshua D., Pittman K., Richardson G., Schou M., Lowe S., Copeman M., De Abreu Lourenco R., Lynch K. (2004). Bisphosphonate infusions: Patient preference, safety and clinic use. Support. Care Cancer.

[6] (2022). Novartis, ZOMETA® (Zoledronic Acid) Injection, for Intravenous Use: Prescribing Information.

[7] Dixon R.B., Tricker N.D., Garetto L.P. (1997). Bone turnover in elderly canine mandible and tibia. J. Dent. Res..

[8] Kim J.E., Yoo S., Choi S.C. (2020). Several issues regarding the diagnostic imaging of medication-related osteonecrosis of the jaw. Imaging Sci. Dent..

[9] Baron R., Ferrari S., Russell R.G. (2010). Denosumab and Bisphosphonates: Different Mechanisms of Action and Effects. Bone.

[10] Ruggiero S.L., Dodson T.B., Aghaloo T., Carlson E.R., Ward B.B., Kademani D. (2022). American Association of Oral and Maxillofacial Surgeons’ Position Paper on Medication-Related Osteonecrosis of the Jaws-2022 Update. J. Oral Maxillofac. Surg..

[11] Lorenzo S.D., Trapassi A., Corradino B., Cordova A. (2013). Histology of the oral mucosa in patients with BRONJ at III Stage: A microscopic study proves the unsuitability of local mucosal flaps. J. Clin. Med. Res..

[12] Curi M.M., Cossolin G.S., Koga D.H., Zrdetto C., Christianini S., Feher O., Cardoso C.L., dos Santos M.O. (2011). Bisphosphonate-related osteonecrosis of the jaws—An initial case series report of treatment combining partial bone resection and autologous platelet-rich plasma. J. Oral Maxillofac. Surg..

[13] Bracher A.I., Vig N., Burkhard J.P., Schaller B., Schlittler F. (2021). The application of platelet rich fibrin in patients presenting with osteonecrosis of the jaw: A systematic literature review. Adv. Oral Maxillofac. Surg..

[14] Parise G.K., Costa B.N., Nogueira M.L., Sassi L.M., Schussel J.L. (2023). Efficacy of fibrin-rich platelets and leukocytes (L-PRF) in tissue repair in surgical oral procedures in patients using zoledronic acid-case-control study. Oral Maxillofac. Surg..

[15] Maracineanu R., Tudor A., Hum I., Urtila F., Streian F., Talpos-Niculescu S., Motoc M. (2025). Platelet-Rich Fibrin in MRONJ management: A prospective comparative study on its effectiveness in prevention and treatment. Medicina.

[16] Agrillo A., Filiaci F., Ramieri V., Riccardi E., Quarato D., Rinna C., Gennaro P., Cascino F., Mitro V., Ungari C. (2012). Bisphosphonate-related osteonecrosis of the jaw (BRONJ): 5 year experience in the treatment of 131 cases with ozone therapy. Eur. Rev. Med. Pharmacol. Sci..

[17] Vescovi P., Manfredi M., Merigo E., Guidotti R., Meleti M., Pedrazzi G., Fornaini C., Bonanini M., Ferri T., Nammour S. (2012). Early surgical laser-assisted management of bisphosphonate-related osteonecrosis of the jaws (BRONJ): A retrospective analysis of 101 treated sites with long-term follow-up. Photomed. Laser Surg..

[18] Cella L., Oppici A., Arbasi M., Moretto M., Piepoli M., Vallisa D., Zangrandi A., Di Nunzio C., Cavanna L. (2011). Autologous bone marrow stem cell intralesional transplantation repairing bisphosphonate related osteonecrosis of the jaw. Head Face Med..

[19] Zhao R., Yang R., Cooper P.R., Khurshid Z., Shavandi A., Ratnayake J. (2021). Bone Grafts and Substitutes in Dentistry: A Review of Current Trends and Developments. Molecules.

[20] Shi K., Xu Y., Chen Z., Shan L., Zhao J., Sun Z., He Z., Wang L., Zheng Y. (2025). Preventive effect of platelet-rich plasma/Bio-Oss granules composite on medication-related osteonecrosis of the jaw in a rat model. J. Dent..

[21] Paulo S., Laranjo M., Abrantes A.M., Casalta-Lopes J., Santos K., Gonçalves A.C., Paula A.B., Marto C.M., Sarmento-Ribeiro A.B., Carrilho E. (2019). Synthetic Calcium Phosphate Ceramics as a Potential Treatment for Bisphosphonate-Related Osteonecrosis of the Jaw. Materials.

[22] Da Silva J.R., Balbas M.C.M., Corrêa C.Á., Zanela M., Okamoto R., Pereira R.D.S., Homsi N., Hochuli-Vieira E. (2022). The Role of Bone Grafts in Preventing Medication-Related Osteonecrosis of the Jaw: Histomorphometric, Immunohistochemical, and Clinical Evaluation in Animal Model. Craniomaxillofacial Trauma Reconstr..

[23] Eguchi T., Kanai I., Basugi A., Miyata Y., Inoue M., Hamada Y. (2017). The assessment of surgical and non-surgical treatment of stage II medication-related osteonecrosis of the jaw. Med. Oral Patol. Oral Cir. Bucal.

[24] Osaka R., Kato H., Hamada Y., Fujimoto Y., Mizusawa N., Watanabe D., Kaneko A. (2021). Clinicostatistical Analyses of Medication-Related Osteonecrosis of the Jaws (MRONJ): Evaluation of the Treatment Method and Prognosis. Oral Sci. Int..

[25] El-Rabbany M., Lam D.K., Shah P.S., Azarpazhooh A. (2019). Surgical management of medication-related osteonecrosis of the jaw is associated with improved disease resolution: A retrospective cohort study. J. Oral Maxillofac. Surg..

[26] Chen S., Ren H., He Y., An J., Zhang Y. (2021). Recurrence-Related Factors of Medication-Related Osteonecrosis of the Jaw: A Five-Year Experience. J. Oral Maxillofac. Surg..

[27] Boffano P., Agnone A.M., Neirotti F., Bonfiglio R., Brucoli M., Ruslin M., Durković A., Milosavljević M., Konstantinovic V., Rodríguez J.C.V. (2024). Epidemiology, etiopathogenesis, and management of MRONJ: A European multicenter study. J. Stomatol. Oral Maxillofac. Surg..

[28] Choi N.R., Lee J.H., Park J.Y., Hwang D.S. (2020). Surgical Treatment of Medication-Related Osteonecrosis of the Jaw: A Retrospective Study. Int. J. Environ. Res. Public Health.

[29] Ruan H.J., Li M.Y., Zhang Z.Y., Ma H.L., He Y. (2024). Medication-related osteonecrosis of the jaw: A retrospective single center study of recurrence-related factors after surgical treatment. Clin. Oral Investig..

[30] de Oliveira Ruellas A.M., Peruzzo D.C., Napimoga M.H. (2017). Managing bisphosphonate-related osteonecrosis of the jaws with xenografts: A case report. Clin. Case Rep..

[31] Yoon S.-Y. (2019). Two Bone Graft Cases Using Xenograft Materials in Complicated Patients with Severe Alveolar Bone Resorption. *Hiossen Implant Can*. https://www.hiossenimplantcanada.ca/two-bone-graft-cases-using-xenograft-materials-in-complicated-patients-with-severe-alveolar-bone-resorption/.

